# Alteration of microbial composition in the skin and blood in vasculitis

**DOI:** 10.1038/s41598-023-42307-7

**Published:** 2023-09-15

**Authors:** Ryujin Miyata, Chie Miyabe, Hiroya Oki, Daisuke Motooka, Shota Nakamura, Yoshishige Miyabe, Yuko Takenaka, Yasuko Fukuya, Kazuo Yudo, Naoko Ishiguro

**Affiliations:** 1 Division of Dermatology, Department of Dermatology, Tokyo Women’s Medical University, Tokyo, Japan; 2 Department of Frontier Medicine, Institute of Medical Science, St. Marianna University School of Medicine, 2-16-1 Sugao, Kawasaki City, Kanagawa Japan; 3 Department of Infection Metagenomics, Genome Information Research Center, Osaka University Research Institute for Microbial Diseases, Suita, Japan; 4 Department of Immunology and Parasitology, St. Marianna University School of Medicine, Kawasaki, Japan

**Keywords:** Immunological disorders, Infectious diseases, Infectious diseases, Inflammation, Vasculitis syndromes

## Abstract

Vasculitis is a systemic autoimmune disease characterized by leukocyte infiltration into blood vessels. Various microorganisms have been associated with the pathogenesis of vasculitis; however, the causal microbial agents and underlying mechanisms are not fully understood, possibly because of the technical limitations of pathogen detection. In the present study, we characterized the microbiome profile of patients with cutaneous vasculitis using comprehensive metagenome shotgun sequencing. We found that the abundance of the SEN virus was increased in the affected skin and serum of patients with vasculitis compared to healthy donors. In particular, the abundance of SEN virus reads was increased in the sera of patients with cutaneous arteritis. Among the bacteria identified, *Corynebacteriales* was the most differentially associated with vasculitis. Linear discriminant analysis effect size also indicated differences in the microbial taxa between patients with vasculitis and healthy donors. These findings demonstrate that vasculitis is associated with considerable alteration of the microbiome in the blood and skin and suggest a role for the infectious trigger in vasculitis.

## Introduction

Vasculitis is a group of autoimmune diseases that cause various organ disorders, not only in the blood vessels but also in the central nerves, lungs, and kidneys^1^. Corticosteroids and immunosuppressive drugs are commonly used to treat vasculitis. However, the disease is intractable to the treatments in some cases; immunosuppression sometimes results in serious clinical complications, and a proper treatment for these conditions has not been established^2^. Since vasculitis is a relatively rare disease compared to other autoimmune diseases such as rheumatoid arthritis, it has not been studied sufficiently, and its etiology has not been fully elucidated^3^.

The association between vasculitis and various pathogens has been widely reported^4^. For example, immunoglobulin (Ig)A vasculitis is often triggered by a prior infection, such as an upper respiratory tract infection, caused by various microorganisms, including *Streptococcus*, *Staphylococcus aureus*, and varicella-zoster virus^5^. With regard to cutaneous small vessel vasculitis (CSVV), previously reported cause of the disease includes infections (20%), inflammatory conditions (15% to 20%), drug reactions (10% to 15%), or malignancies (5%), and the remaining half of CSVV cases are considered to be idiopathic^6^. We previously showed that a streptococcal antigen, the nephritis-associated plasmin receptor, is deposited in cutaneous vessels in IgA vasculitis, possibly inducing vascular inflammation^7^. Polyarteritis nodosa (PAN) is triggered by hepatitis B virus (HBV) infection^8^, and HBV on the vascular wall has been identified at the site of vasculitis^9^, suggesting that the pathogen may directly induce some types of vasculitis.

Studies on the association between vasculitis and pathogens have been conducted using animal models. For instance, injection of *Candida albicans* water-soluble fraction in mice induces inflammation of the aortic root and coronary arteries^10–13^, as well as in experimental models of vasculitis caused by viral, bacterial, fungal, and other pathogens^4^. Another study attempted to identify the causal microbe of the temporal arteries in patients with giant cell arteritis (GCA), and 16S ribosomal RNA gene sequencing revealed microbiome differences in temporal arteries between GCA and non-GCA. However, no single pathogen that causes GCA has been identified^14^.

With the advent of next-generation sequencing (NGS) technology, researchers have been able to obtain large amounts of data in a single round of operation^15^. NGS methods also enable the detection of microorganisms that are difficult to culture. Currently, most of these investigations use NGS by focusing on targeted amplicon sequencing (16S rRNA gene sequencing), or by sequencing the genetic material present within a sample directly using a PCR-independent approach (shotgun metagenomic sequencing)^16^. As shotgun metagenomic sequencing is PCR-independent, it comprehensively explores microorganisms and leads to the discovery of unknown novel pathogens that cannot be detected using targeted amplicon-based methods^17^. Indeed, a study comparing 16S rRNA gene sequencing with shotgun metagenomic sequencing for taxonomic characterization of gut microbiota showed that 16S rRNA gene sequencing detected only a fraction of the microbiota identified by shotgun sequencing^18^.

Among the various organs involved in vasculitis, the skin is the most privileged tissue because it is easily accessible for physical examinations and biopsies in comparison with other organs^19^. Thus, understanding the pathogenesis of vasculitis by identifying the causal microorganisms in the skin and blood could lead to a new therapeutic approach. In this study, we used NGS to analyze the microbial genome in skin and serum derived from patients with vasculitis.

## Results

### Viral profile of serum and skin tissue in vasculitis and HD (healthy donors)

**Figure 1:**
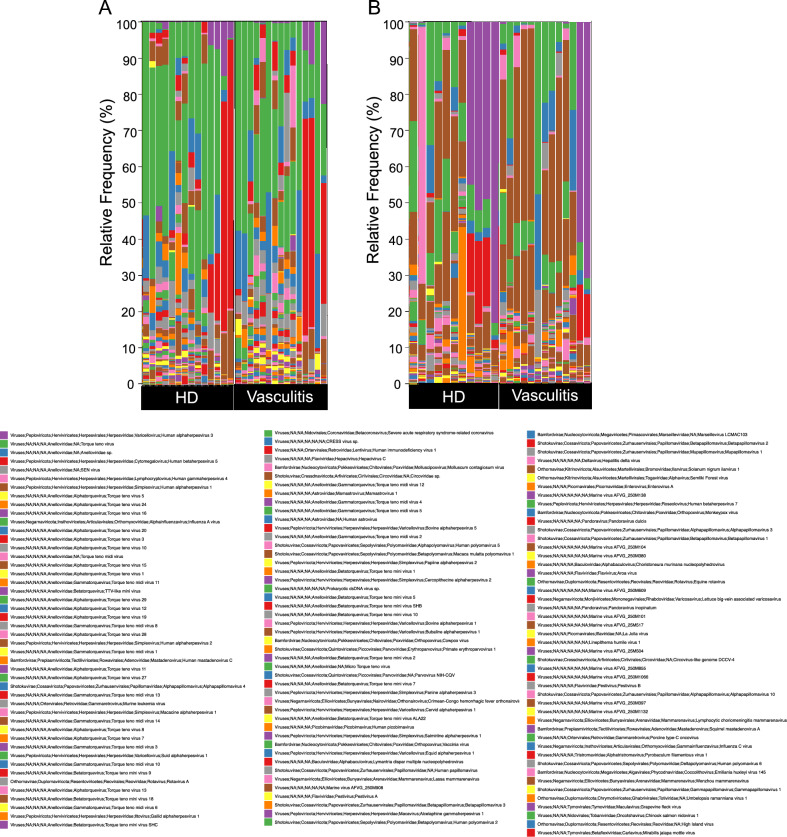
Viral composition in patients with vasculitis and HD. Stacked graphs show the distribution of the relative frequency of viral species in serum (**A**) or skin tissue (**B**).

**Figure 2:**
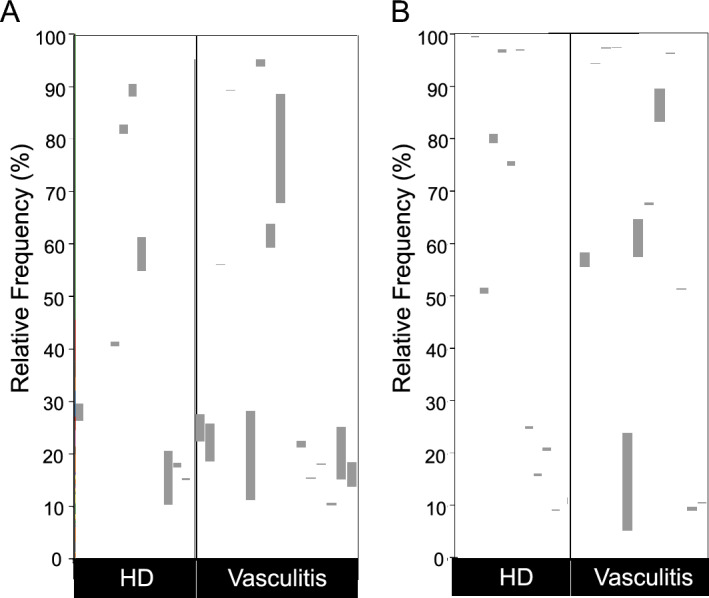
The proportion of SEN virus detected in patients with vasculitis and HD. Purple bar plot indicates relative frequency of SEN virus read counts in serum (**A**) or skin tissue (**B**).

We first analyzed the viral composition in the serum and skin tissue using the Kraken2 software to determine if any specific species were enriched in the samples derived from patients with vasculitis. Interestingly, a large variety of viral species was detected in the serum (Fig. 1A), which is normally considered a sterile body fluid. The skin virome is also composed of many types of viruses (Fig. 1B), and the frequency of *Human alphaherpesvirus 1* detected in the skin appeared to be higher than that in the serum (shown in brown). Among the viruses, the SEN virus (shown in gray) was more frequently detected in serum derived from patients with vasculitis (Fig. 2A), as well as in skin tissue derived from patients with vasculitis (Fig. 2B), than in HD (healthy donors).

The SEN virus has been linked to blood transfusion and is thought to be an important cause of post-transfusion hepatitis^20^. Although there is no reliable evidence that SEN virus infection leads to hepatitis, concurrent infections with the SEN virus, HBV, hepatitis C virus (HCV), or human immunodeficiency virus type 1 have been documented^21^. As HBV and HCV are associated with systemic vasculitis, we sought to investigate the possibility that the presence of the SEN virus influences the disease status of vasculitis.

### SEN virus is abundantly detected in vasculitic skin tissue

**Figure 3:**
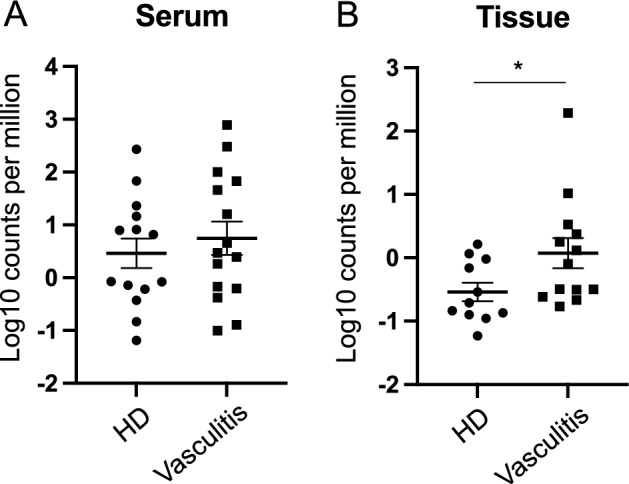
Abundance-normalized read counts (proportion of read counts) of SEN virus in patients with vasculitis and HD. (**A**) Serum read counts of SEN virus in patients with vasculitis and HD. (**B**) Tissue read counts of SEN virus in patients with vasculitis and HD. Values are mean ± SEM. *P < 0.05 vs. HD.

We compared vasculitis patients with HD based on the abundance-normalized read counts (proportion of read counts) of the SEN virus. Although there was no significant difference in serum (Fig. 3A), the read counts of the SEN virus were significantly higher in skin tissue from patients with vasculitis than in HD (Fig. 3B). We also conducted qPCR to compare the SEN virus-positive rates in each serum sample. SEN virus was detected in 13 (76.5%) of the 17 patients with vasculitis and in 2 (40%) of the 5 HD (Table 1). The SEN virus has a tendency to be present in patients with vasculitis compared to those on HD. These results suggest that the SEN virus may be associated with skin symptoms in vasculitis. There was no remarkable correlation between the presence of the SEN virus and liver dysfunction in this patient cohort.

**Table 1:** SEN virus-positive rates for patients with vasculitis and healthy donors.

Patient group	SENV positive rate	P-value^1^
Vasculitis	76.5% (13/17)	0.1603
Healthy donor	40% (2/5)	

^1^Fisher’s exact test. Data are percentage of positive cases (number of positive cases/total number in group).

### Serum viral reads of SEN virus based on patient’s characteristics

**Figure 4:**
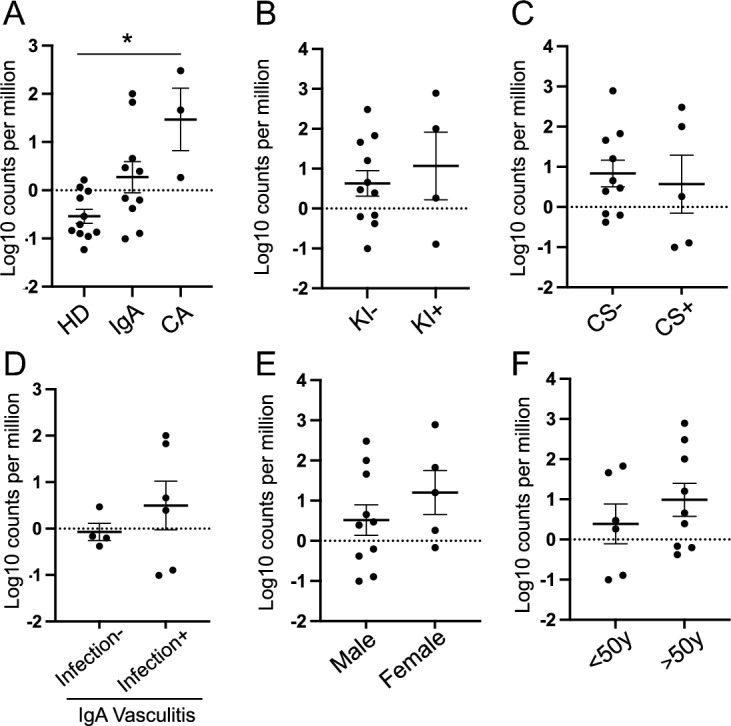
Abundance-normalized serum read counts (proportion of read counts) of SEN virus by patient characteristics. (**A**) Type of vasculitis. (**B**) Kidney involvement. (**C**) Use of corticosteroids. (**D**) Episode of preceding infection in IgA vasculitis. (**E**) Sex. (**F**) Age. Values are mean ± SEM. *P < 0.05 vs. HD.

**Figure 5:**
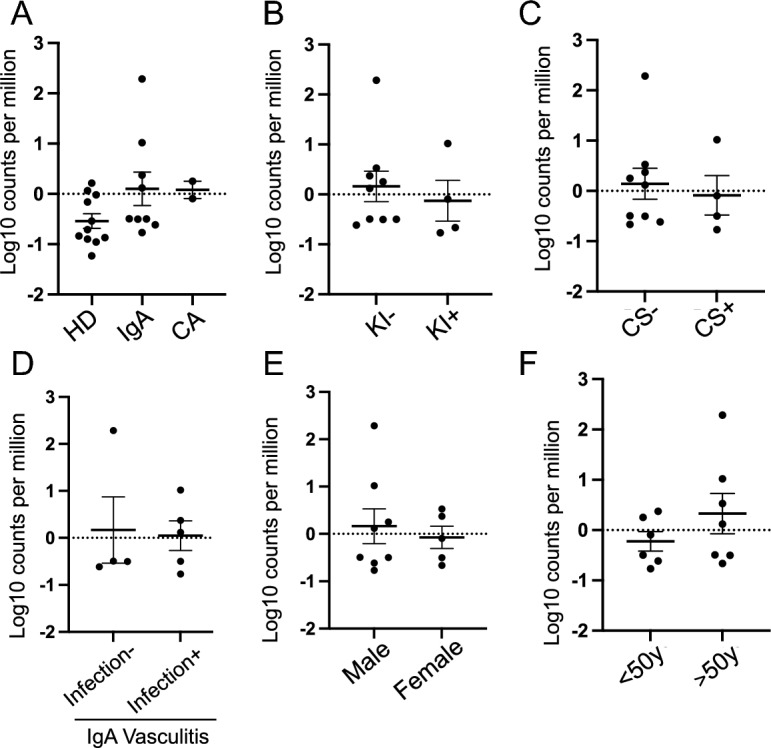
Abundance-normalized tissue read counts (proportion of read counts) of SEN virus by patient characteristics. (**A**) Type of vasculitis. (**B**) Kidney involvement. (**C**) Use of corticosteroids. (**D**) Episode of preceding infection in IgA vasculitis. (**E**) Sex. (**F**) Age. Values are mean ± SEM.

We further explored whether the reads of the SEN virus differed depending on the clinical features of the patients. The proportion of read counts for the SEN virus was significantly higher in patients with CA than in those on HD (Fig. 4A). However, there were no differences with regard to other clinical features, such as kidney involvement, use of corticosteroids, episode of preceding infection in IgA vasculitis, sex, or age in patients with vasculitis (Fig. 4B–F). Viral reads of the SEN virus in the skin tissue were not statistically different according to the clinical features of the patients with vasculitis (Fig. 5A–F). In HD, there was no statistical difference in the viral reads of the SEN virus by sex and age (Fig. S1, Supplementary Information).

### Bacterial composition in serum from patients with vasculitis and HD

**Figure 6:**
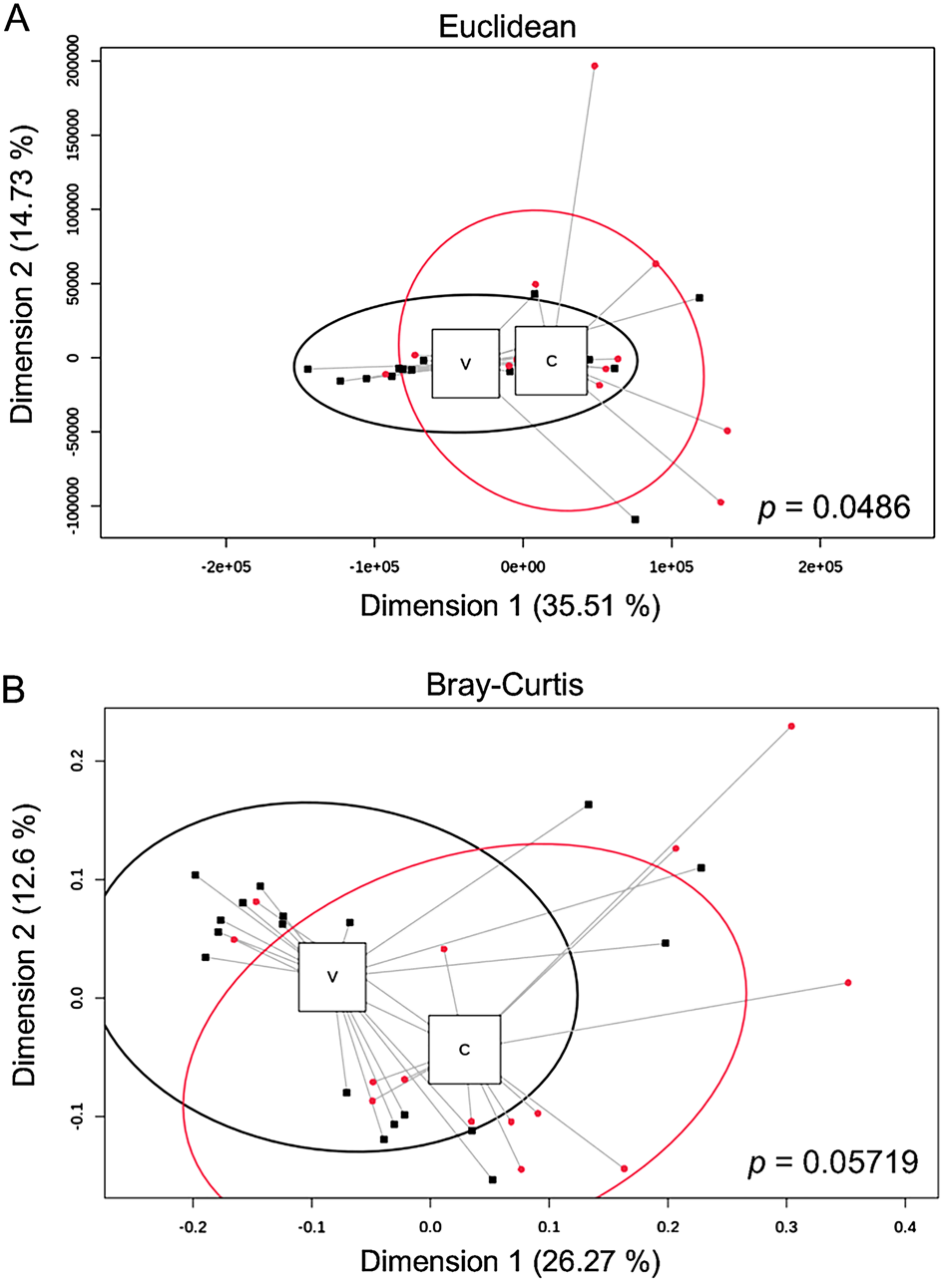
Circulating bacterial profile in patients with vasculitis and HD. Principal component analysis (PCoA) of serum from patients with vasculitis (black) compared to those from HD (red). P values less than 0.05 were considered as statistically significant.

IgA vasculitis is one of the most common types of vasculitis that present cutaneous symptoms. Although the mechanism in which the skin and kidney are involved in IgA vasculitis has not been clarified, numerous studies have linked IgA vasculitis with bacterial infection, particularly with β-hemolytic streptococci, as a possible trigger for the disease^22^. The majority of the patients enrolled in this study had IgA vasculitis. We therefore analyzed the bacterial composition in this cohort to determine whether bacteria were associated with the pathogenesis of vasculitis. The beta diversity of serum samples using PCA based on Euclidean distance showed a tendency toward a difference between vasculitis and HD (PERMANOVA: R^2^ = 0.06465, *p* = 0.0486) (Fig. 6A). There was no significant difference between the vasculitis and HD groups in terms of the Bray–Curtis distance (PERMANOVA: R^2^ = 0.05241, *p* = 0.05719) (Fig. 6B).

**Figure 7:**
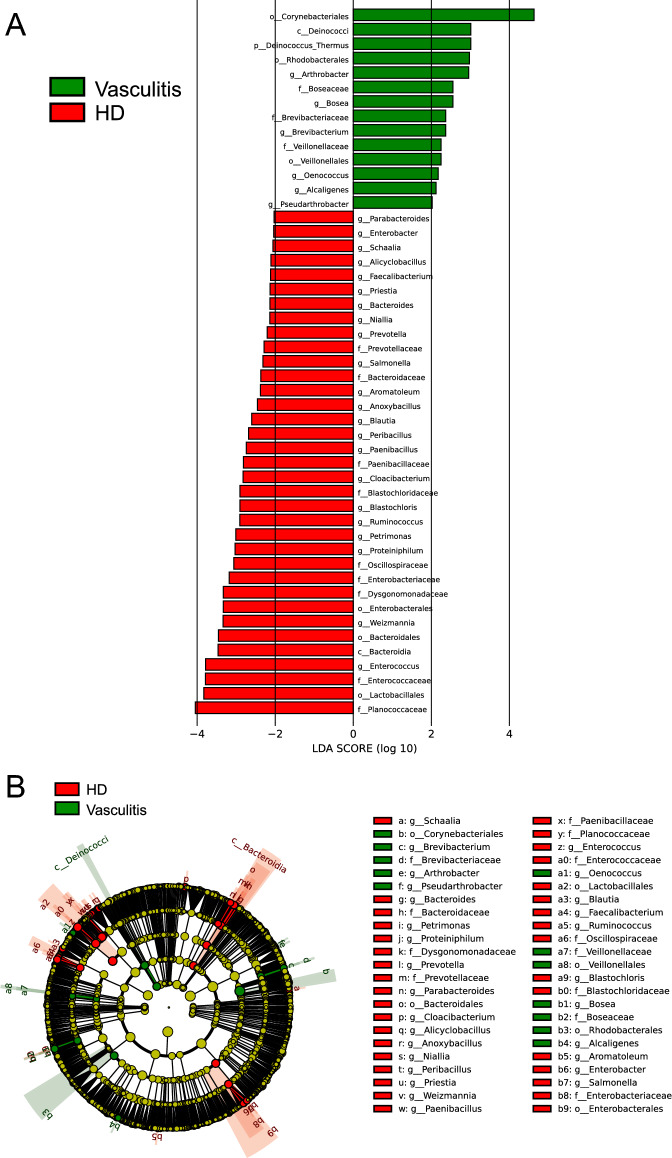
LDA effect size (LEfSe) analysis. (**A**) The bar plots represent the significantly differential taxa between vasculitis (green) or HD (red) (LDA score > 2). (**B**) Cladograms showing the taxa most differentially associated with vasculitis (green) or HD (red). Circle sizes in the cladogram plot are proportional to bacterial abundance. The circles represent, going from the inner to outer circle: phyla, genera, classes, order, and families. Yellow nodes represent species with no significant difference.

We also analyzed serum bacterial taxa using LDA effect size (LEfSe) to detect differences between patients with vasculitis and HD. The bar plot shows linear discriminant analysis (LDA) scores of microbial taxa, with significant differences between the vasculitis and HD groups (Fig. 7A). The cladogram also indicated differences in microbial taxa between patients with vasculitis and those on HD (Fig. 7B). The bacterial read that was remarkably elevated in the vasculitis serum was from *Corynebacteriales*.

## Discussion

The ability of leukocytes to enter tissues in response to immune stimuli is a central feature of the host defense^23^. On the other hand, excessively activated leukocytes, including lymphocytes, macrophages, and neutrophils, damage the vessels and aggravate inflammation^12^. While the etiology of vasculitis remains incompletely understood, an increasing number of animal and human studies have suggested that it is associated with a variety of pathogenic agents including bacteria, viruses, and fungi^4^. Previous studies have indicated that viruses relate to small-vessel vasculitis in many cases, whereas bacterial infections cause vasculitis in all sizes of vessels^24^.

In the present study, we utilized comprehensive metagenomic shotgun sequencing to demonstrate that the composition of viruses and bacteria in the serum and skin tissue is altered in vasculitis. Specifically, the frequency of the SEN virus detected in the skin tissue is elevated in vasculitis. In addition, the SEN virus is more likely to be present in the serum of patients with cutaneous arteritis than in those with IgA vasculitis.

The SEN virus is a blood-borne single-stranded circular DNA virus discovered in 1999^21^. The prevalence of the SEN virus has been reported to be 10% in Japan^25^, with a higher incidence among patients with liver diseases in comparison with the general population. Among the nine different genetic variants of the SEN virus (A to I), the most predominant genotypes are SEN virus D and H, which are typically found in patients with non-A-E hepatitis^26^. HBV is associated with cryoglobulinemic small-vessel vasculitis and polyarteritis nodosa (PAN), and the relationship between PAN and HBV has been often reported^4^. Approximately 5% of patients with HCV-associated mixed cryoglobulinemia develop cryoglobulinemic vasculitis, as a consequence of the deposition of immune complex (IC) in small vessels. Although we were not able to histologically verify the presence of the SEN virus in the skin vessels owing to a lack of available SEN virus-specific antibodies, it is possible that direct injury of the vessel wall or IC deposition in the vessel wall induces vascular inflammation, similar to other viruses. In addition, IgA vasculitis might be a more heterogeneous condition than CA, based on the finding that the frequency of the SEN virus was higher in serum samples from CA, but not in those from IgA, compared to HD.

In the present study, we examined the bacterial composition of patients with vasculitis on HD. No remarkable changes in bacterial diversity were detected in the sera of patients with vasculitis and HD. However, the abundance of bacterial species was considerably different between the sera derived from patients with vasculitis and HD. Among the bacteria identified in the serum, *Corynebacteriales* were the most differentially associated with vasculitis. The genus *Corynebacterium* is a taxon of coryneform gram-positive bacilli or coccobacilli that colonize the mucous membranes and skin in humans^27^. *Corynebacterium* is usually regarded as a clinically non-significant contaminant in cultures^28^, but is sometimes reported in the context of autoimmune diseases. A previous study suggested that *Corynebacterium 1* in the gut was associated with disease duration, and serum IL-1β levels in patients with vitiligo^29^. Other studies have implicated that the presence of *Corynebacterium* in the nasopharyngeal microbiome^30^ and peripheral blood leukocytes^31^ relates to Kawasaki disease. In the present study, although no bacteria already known to be associated with IgA vasculitis were detected, such as *Staphylococcus* and *Streptococcus species*, *Corynebacterium* species may serve as novel pathogenic candidate microbes for vasculitis.

Some limitations in this study need to be acknowledged. First, we detected numerous microorganisms using metagenome sequences; however, contamination in the blood or skin should always be considered. Although we removed the epidermis before gene extraction to exclude resident skin flora, the presence of *Corynebacterium* in the cutaneous vessels should be further verified as *Corynebacterium* is one of the major skin-resident bacteria^32^. Second, the small sample size and limited types of vasculitis may have affected the results. Third, we did not assess whether the microorganisms were truly pathogenic or were present only in the blood or skin tissue. Further studies using animal models should provide important insights into the pathogenic role of microbiome alterations in vasculitis.

In conclusion, we found specific alterations in the microbiome in cutaneous vasculitis and identified new candidates for the pathogenic microbe using metagenomic shotgun analysis. These alterations of microbial composition may act as environmental triggers or potentially aggravating factors for vasculitis. Further studies are required to verify the importance of these pathogens on the initiation and progression of vasculitis.

## Materials and methods

### Study population

Blood and skin tissue samples were collected from 18 patients with vasculitis who visited our division between 2020 and 2022 and 14 HD. Participants were divided into patients diagnosed with cutaneous arthritis (CA; n = 4), IgA vasculitis (n = 12), microscopic polyangiitis (MPA; n = 1), and cryoglobulinemic vasculitis (n = 1). The authors had access to information that could identify individual participants during or after data collection.

Clinical information is shown in Table 2. There were no significant differences between the patients with vasculitis and those on HD with regard to age or sex. One patient with IgA vasculitis had a history of HBV virus infection. Skin biopsies were taken from the active cutaneous vasculitis lesions in lower legs, except one case of IgA vasculitis taken from the knee. The experimental protocol was approved by the Ethics Committee of Tokyo Women’s Medical University (approval number: 5700) and all subjects provided written informed consent according to the principles of the Declaration of Helsinki.

**Table 2:** Characteristics of the patients with vasculitis and the healthy donors.

	IgA (n = 12)	CA (n = 4)	Other (n = 2)	HD (n = 14)	P-value^1^
Age, years: mean (+ /−SD)	57 (18)	42 (19)	56 (2)	60 (14)	0.44
Sex, females: number (%)	3 (25)	2 (50)	2 (100)	7 (50)	0.72
CS treatment: number (%)	3 (25)	2 (50)	0 (0)		
Kidney involvement: number (%)	4 (33)	0 (0)	0 (0)	–	–
Abdominal symptoms: number (%)	3 (25)	0 (0)	0 (0)	–	–
Neurological symptoms: number (%)	0 (0)	1 (25)	1 (50)	–	–
C-reactive protein (mg/l): mean (+ /−SD)	1.06 (0.9)	0.46 (0.6)	1.03 (0.06)	–	–
Episode of infection: number (%)	8 (75)	0 (0)	0 (0)	–	–

*IgA* IgA vasculitis, *CA* cutaneous arteritis, *HD* healthy donors, *CS* corticosteroids (prednisone), *SD* standard deviation, Analysis: Student’s t-test or Fisher’s exact test.

^1^HD versus all patients with vasculitis.

### DNA and RNA extraction from skin tissue and blood samples

Skin tissue samples were cut into small pieces, vortexed with Phosphate-buffered saline (PBS), and centrifuged to collect the supernatant. The QIAamp MinElute Virus Spin Kit (Qiagen, Hilden, Germany) was used for DNA and RNA extraction from supernatant fractions and blood samples. The quantities of the collected DNA and RNA were measured using a Qubit fluorometer (Thermo Fisher Scientific).

### Metagenomic shotgun sequencing

The extracted RNA was converted into double-stranded copy (c)DNA using the ProtoScript II First Strand cDNA Synthesis Kit (New England Biolabs, Ipswich, MA, USA), NEBNext Ultra II Non-Directional RNA Second Strand Synthesis Module (New England Biolabs), and Random Primer 6 (random hexanucleotides; New England Biolabs). The obtained double-stranded (ds)DNA and DNA samples were subsequently converted to Illumina TruSeq-compatible libraries using Twist Library Preparation Enzymatic Fragmentation Kit (Twist Biosciences, CA, USA) and Twist Universal Adapter System-TruSeq Compatible (Twist Biosciences). We performed a single hybridization capture for each sample and enrichment using the Twist Comprehensive Viral Research Panel (Twist Biosciences). All libraries were converted into libraries for the DNBSEQ using the MGIEasy Universal Library Conversion Kit (App-A). Sequencing was performed using the DNBSEQ-G400RS High-throughput Sequencing Kit (MGI Tech, Tokyo, Japan) in 100-base paired-end mode. Each read was subjected to KRAKEN2 analysis against the PlusPFP database containing archaea, bacteria, viral, plasmid, human, UniVec_core, protozoa, fungi, and plant^33,34^.

### Extraction of serum DNA and quantitative real-time polymerase chain reaction

DNA extraction from 200 μl of serum was carried out with the Plasma/Serum Cell-free Circulating DNA Purification Mini Kit (Norgen, Thorold, Canada) as per protocol. Quantitative polymerase chain reactions (qPCR) in the presence of SYBR Green (PowerUp SYBR Green Master Mix, Applied biosystems, MA, USA) were performed with a QuantStudio 3 (Thermo Fisher Scientific, MA, USA) using the following primers: AI, 5’- TWCYCMAACGACCAGCTAGACCT—3’ (forward) and 5’- GTTTGTGGTGAGGAGAACGGA—3’ (reverse)^35^. Cycle threshold of less than 35 defined a positive result.

### Statistical analysis

Data were analyzed using Prism 9 (GraphPad Software), and results were expressed as the mean ± SEM. Values between the two groups were compared using an unpaired 2-tailed Student’s t-test. *P* values for multiple groups were calculated using ordinary one-way ANOVA with Dunnett’s post hoc test. *P* value for the contingency table was calculated using Fisher's exact test. A value of *P* < 0.05 defined the presence of a statistically significant difference.

### Supplementary Information

Supplementary Figure S1.

## Data Availability

The data that support the findings of this study are available from the corresponding author, [CM], upon reasonable request.
