# Supplementary material for: Alteration of microbial composition in the skin and blood in vasculitis
Source: Sci Rep. 2023 Sep 15;13:15317. doi: 10.1038/s41598-023-42307-7 (PMC10504252; doi:10.1038/s41598-023-42307-7)
Supplement: Supplementary file 1 — Supplementary Figure S1. [file 41598_2023_42307_MOESM1_ESM.pdf]

Supplementary Information

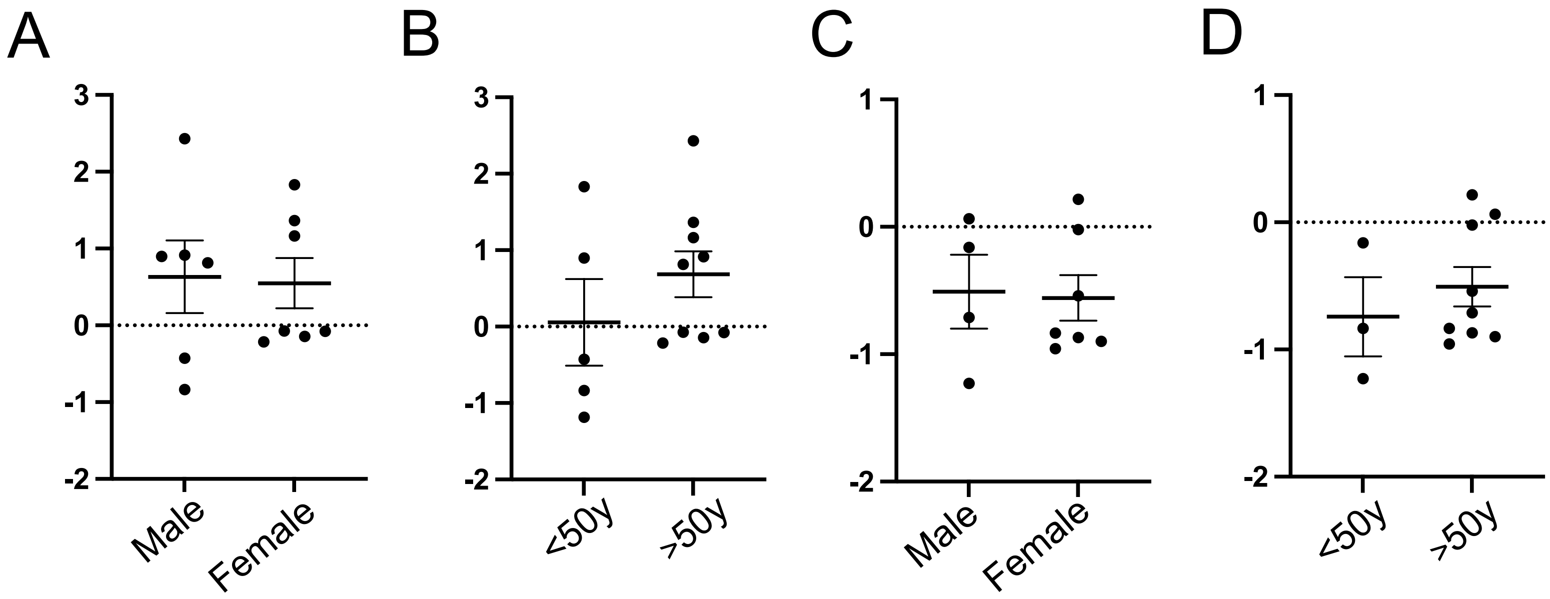

Figure S1. Abundance-normalized tissue read counts (proportion of read counts) of SEN virus by the characteristics of the healthy donors. (A) Serum, sex. (B) Serum, age. (C) Tissue, sex. (D) Tissue, age.
